# Reaction Time and Incident Cancer: 25 Years of Follow-Up of Study Members in the UK Health and Lifestyle Survey

**DOI:** 10.1371/journal.pone.0095054

**Published:** 2014-04-18

**Authors:** Beverly A. Roberts, Ian J. Deary, Dominika Dykiert, Geoff Der, G. David Batty

**Affiliations:** 1 Centre for Cognitive Ageing and Cognitive Epidemiology, University of Edinburgh, Edinburgh, United Kingdom; 2 Medical Research Council Public and Health Sciences Unit, University of Glasgow, Glasgow, United Kingdom; 3 Department of Epidemiology and Public Health, University College London, London, United Kingdom; Kagoshima University Graduate School of Medical and Dental Sciences, Japan

## Abstract

**Objectives:**

To investigate the association of reaction time with cancer incidence.

**Methods:**

6900 individuals aged 18 to 94 years who participated in the UK Health and Lifestyle Survey in 1984/1985 and were followed for a cancer registration for 25 years.

**Results:**

Disease surveillance gave rise to 1015 cancer events from all sites. In general, there was essentially no clear pattern of association for either simple or choice reaction time with cancer of all sites combined, nor specific malignancies. However, selected associations were found for lung cancer, colorectal cancer and skin cancer.

**Conclusions:**

In the present study, reaction time and its components were not generally related to cancer risk.

## Introduction

Higher cognitive scores as measured in childhood, early adulthood, midlife and older age are related to a lower risk of total mortality and selected health outcomes [1]–[6] including psychiatric illness [7]–[9], injury [10], [11] and, in particular, cardiovascular disease [5], [6]. Some of these relationships may be explained by the tendency for higher IQ scoring individuals to have more favourable levels of smoking, physical activity, diet, obesity, blood pressure, and socioeconomic status [12]–[17].

Some of the risk factors for cardiovascular disease (CVD) (e.g. smoking, obesity, physical inactivity which are known to be associated with IQ as stated above) are also those for selected cancers including carcinoma of the lung, prostate, breast [in women], and bowel [18], [19]. Therefore, an IQ-cancer relation would be anticipated. However, few studies have examined the association between cognition and cancer incidence, and those that have reveal contradictory findings. Thus, while some investigators have found null results for IQ in relation to a range of specific cancers [3], [21], particularly after full adjustment for confounders [20], others have found that higher cognition appears to confer protection. [22], [23].

Importantly, in these cancer-cognition studies, cognitive ability was ascertained using standard tests of intelligence which are sometimes seen as being culturally biased. Reaction time has be used as a measure of brain processing speed and has been found to be moderately inversely correlated with general cognitive ability [24]. Furthermore, when examining the association between psychometric intelligence and reaction time with mortality, Deary and Der [24] also found that after adjusting for reaction time, the association between psychometric intelligence and mortality was attenuated. Thus, reaction time, or the brain's processing speed, can be seen as a measure that correlates with and accounts for some of the health-related variance of cognitive performance, with shorter (faster) reaction times being apparent in people with higher cognitive ability [24]. Reaction time may therefore be regarded as a relatively culture-reduced measure of cognitive function.

Using the UK Health and Lifestyle Survey (HALS) we have previously shown that reaction time is associated with all-cause and cause specific mortality [2], [3] such that lower rates of these outcomes are apparent in people with shorter reaction time. We now investigate whether cognition, as measured using reaction time, is related to cancer incidence in this study. To our knowledge, this is the first examination of the cognition-cancer relation in which reaction time is the measure of cognition.

## Methods

### Participants

Participants were drawn from the HALS, a nationally representative, cross-sectional survey of individuals aged over 18 years in England, Wales and Scotland. Full details of data collection have been described elsewhere [25]. In brief, in 1984/1985, 12,254 addresses were randomly selected from the English, Welsh and Scottish Electoral Registers, which yielded interviews with 9003 (73.5%) individuals. Physiological and cognitive measurements were complete for 7414 individuals during a second home visit. The same procedures were repeated at HALS2 in 1991/1992 (data not included here). HALS participants have been followed up to ascertain mortality and cancer incidence, with the latest cancer follow up being until 30^th^ June 2009. Up to 30^th^ June 2009, 1468 of the 9003 respondents have been coded for cancer incidence (malignant and benign). HALS data is freely available to download from the UK Economic and Social Data Service (www.esdc.ac.uk).

### Reaction time

Simple and four-choice reaction time were measured using a portable electronic device consisting of a small LCD screen and 5 buttons numbered 1,2,0,3,4 from left to right (see [2] for a figure of the device). Simple reaction time (SRT) was the time taken to press the ‘0’ key after a ‘0’ stimulus appears on the screen. There were eight practice trials and 20 test trials. Choice reaction time (CRT) was the time taken to press the correct one of four keys corresponding to the presentation of one of four digits on the display. Eight practice trials were followed by 40 test trials (ten each of the four digits in random order). Both mean reaction time score and variability in reaction time performance was used as measures. Reaction time variabilities are the standard deviation of the reaction time trials: of 20 for simple reaction time and 40 for choice reaction time. A low value for the reaction time indices represents faster speed or less variability signifying better performance.

### Cancer

Information on cancer registrations is provided by the NHS central registry. For the present analysis, only primary malignant cancers were considered. Data on cancer incidence registrations were grouped according to ICD-9 classifications into all cancer, smoking-related cancers [26], non smoking-related cancers, lung cancer, colorectal cancer, skin cancer, male prostate cancer and female breast cancer. Those who received a cancer diagnosis prior to and within year 1 of the study (September 1984-August 1985) were removed.

### Covariates

Age in years was included in the models as a continuous variable. Occupational social class was based on the Registrar General's occupational social class categories (Office of Population Censuses and Surveys, 1980) using the current or usual occupation of the head of the household. It comprises six categories which include professional, managerial and technical, skilled (non-manual), skilled (manual), partly skilled and unskilled. Alcohol consumption (always non-drinker, very special occasion drinker, occasional drinker and regular drinker) and current smoking status (never smoked, current regular cigarette smoker, occasional cigarette smoker and ex-regular cigarette smoker) were based on standard enquiries and categorisation. Furthermore, as long duration of smoking is known to increase colorectal cancer risk, we included pack years of smoking as an additional confounder in this one model. Occupational and leisure physical activity was also recorded. Such activities include work-associated physical activity, walking, housework, gardening, and a variety of sports. For each activity, the total duration in minutes, and number of occasions per fortnight were recorded, in addition to its level of intensity. This information gave four variables: minutes spent doing vigorous activity, minutes spent doing non-vigorous activity, occasions spent doing vigorous activity, and occasions spent doing non-vigorous activity.

### Statistical analysis

Multivariable analysis was carried out using Cox's proportional hazard regression in SAS.[27] This was used to calculate hazard ratios (with accompanying 95% confidence intervals) for the proportionate change in cancer incidence risk for each standard deviation difference in reaction time measures. Here, an increase in the reaction time value indicates disadvantage in cognition while a decrease indicates advantage.

For the survival analysis, entry in to the study began on the date of the baseline survey in 1984/85 for all participants. Calculations of person-years-at-risk was then based on 30^th^ June 2009 (end of cancer follow-up) for those with no cancer, date of death for those who died cancer free, and date of first cancer registration for those who developed a cancer. Within HALS there was no data on the exact date of migration for those lost to follow-up (N = 55). Therefore, so as not to overestimate survival time for these individuals by censoring at 30^th^ June 2009, we censored at the baseline survey date for those who did not attend the HALS2 follow-up study (1991/1992) and censored those who did attend the HALS2 follow-up at that time.

## Results

Only participants who had complete data with respect to reaction time measures, cancer registrations (malignant cancer only) and all covariates were included in the analysis. This yielded a final sample of 6900 individuals (3809 women) with a mean age of 44.9 years and age range of 18–94 years.

During a mean of 24.6 years of follow-up there were 1015 (14.7%) cancer events in the 6900 participants. Of these, 467 (46.0%) were smoking-related cancers (lung, oesophagus, larynx, pharynx, pancreas, bladder, sinuses, stomach, liver, kidney, cervix, bowel, ovarian, and myeloid leukaemia) [26], 548 (54.0%) were non smoking-related cancers (all other cancers), 151 (14.9%) were lung cancer, 109 (10.7%) were colorectal cancer, 164 (16.1%) were skin cancer, 99 (N = 3091 men only, 3.2%) were prostate cancer, and 142 (N = 3809 women only, 3.7%) were female breast cancer.

Examining the multivariate association between simple and choice reaction time mean and variability, and cancer diagnoses, none of the hazard ratios for all-cancer incidence were statistically significant at conventional levels of significance in any of the four multivariate models. In models 1–4, null results were also generally found for all cancer specific sites. Exceptions to this were CRT mean and lung cancer (model 1: HR = 1.20 (1.02, 1.40)), and SRT variability with skin cancer (model 1: HR = 1.20 (1.05, 1.38)) and colorectal cancer (model 2: HR = 1.20 (1.01, 1.42)) (tables 1–4). The models were further tested by including a quadratic term for age. This did not alter the results. There was also no statistically significant sex by age interactions.

**Table 1:** Hazard ratios and 95% confidence intervals for the relation of a one standard deviation increase (disadvantage) with cancer incidence (N = 6900).

	Model 1 (age and sex)	Model 2 (model 1 + social class)	Model 3 (model 1 + smoking, alcohol, physical activity)	Model 4 (all covariates)
**All cancer (cases = 1015)**				
Simple reaction time mean	1.02 (0.97, 1.08)	1.02 (0.96, 1.08)	1.02 (0.96, 1.08)	1.02 (0.96, 1.08)
Simple reaction time variability	1.06 (0.99, 1.12)	1.05 (0.99, 1.12)	1.05 (0.98, 1.12)	1.05 (0.98, 1.11)
Choice reaction time mean	1.03 (0.96, 1.11)	1.03 (0.95, 1.11)	1.02 (0.98, 1.03)	1.02 (0.94, 1.07)
Choice reaction time variability	1.01 (0.96, 1.06)	1.00 (0.95, 1.06)	1.00 (0.95, 1.06)	1.00 (0.95, 1.05)
**All smoking-related cancers** * **(cases = 467)**				
Simple reaction time mean	1.04 (0.96, 1.12)	1.03 (0.95, 1.12)	1.03 (0.95, 1.12)	1.03 (0.95, 1.12)
Simple reaction time variability	1.04 (0.96, 1.14)	1.03 (0.95, 1.13)	1.02 (0.93, 1.12)	1.02 (0.93, 1.11)
Choice reaction time mean	1.11 (0.99, 1.23)	1.09 (0.98, 1.21)	1.06 (0.95, 1.19)	1.05 (0.94, 1.18)
Choice reaction time variability	1.02 (0.94, 1.09)	1.01 (0.94, 1.09)	1.00 (0.92, 1.08)	0.99 (0.91, 1.08)

* Smoking-related cancers include lung, oesophagus, larynx, pharynx, pancreas, bladder, sinuses, stomach, liver, kidney, cervix, bowel, ovarian, and myeloid leukaemia [26].

**Table 2:** Hazard ratios and 95% confidence intervals for the relation of a one standard deviation increase (disadvantage) with cancer incidence (N = 6900).

	Model 1 (age and sex)	Model 2 (model 1 + social class)	Model 3 (model 1 + smoking, alcohol, physical activity)	Model 4 (all covariates)
All non smoking-related cancers* (cases = 548)				
Simple reaction time mean	1.01 (0.93, 1.09)	1.01 (0.93, 1.09)	1.01 (0.93, 1.09)	1.01 (0.93, 1.09)
Simple reaction time variability	1.07 (0.98, 1.16)	1.07 (0.98, 1.17)	1.08 (0.99, 1.17)	1.08 (0.99, 1.17)
Choice reaction time mean	0.97 (0.87, 1.08)	0.97 (0.87, 1.08)	0.99 (0.88, 1.10)	0.98 (0.88, 1.10)
Choice reaction time variability	1.00 (0.93, 1.08)	1.00 (0.93, 1.08)	1.01 (0.94, 1.08)	1.01 (0.93, 1.08)
**Lung cancer (cases = 151)**				
Simple reaction time mean	1.01 (0.89, 1.16)	1.00 (0.87, 1.15)	1.06 (0.95, 1.18)	0.99 (0.86, 1.15)
Simple reaction time variability	1.03 (0.88, 1.19)	1.00 (0.86, 1.17)	0.99 (0.85, 1.15)	0.98 (0.84, 1.14)
Choice reaction time mean	1.20 (1.02, 1.40)	1.16 (0.99, 1.37)	1.12 (0.94, 1.33)	1.10 (0.92, 1.32)
Choice reaction time variability	1.07 (0.96, 1.19)	1.06 (0.95, 1.18)	1.05 (0.92, 0.19)	1.04 (0.92, 1.18)

* All other cancers not related to smoking.

**Table 3:** Hazard ratios and 95% confidence intervals for the relation of a one standard deviation increase (disadvantage) with cancer incidence (N = 6900).

	Model 1 (age and sex)	Model 2 (model 1 + social class)	Model 3 (model 1 + smoking, alcohol, physical activity)	Model 4 (all covariates)
**Colorectal cancer (cases = 109)**				
Simple reaction time mean	1.08 (0.94, 1.25)	1.10 (0.95, 1.26)	1.07* (0.93, 1.24)	1.08 (0.93, 1.24)
Simple reaction time variability	1.17 (0.99, 1.39)	1.20 (1.01, 1.42)	1.17* (0.99, 1.39)	1.19 (0.99, 1.42)
Choice reaction time mean	1.13 (0.92, 1.40)	1.17 (0.95, 1.44)	1.13* (0.92, 1.40)	1.16 (0.94, 1.43)
Choice reaction time variability	0.98 (0.83, 1.17)	0.99 (0.84, 1.18)	0.99* (0.84, 1.18)	1.00 (0.84, 1.19)
**Skin cancer (cases = 99)**				
Simple reaction time mean	1.10 (0.97, 1.24)	1.10 (0.97, 1.24)	1.10 (0.97, 1.24)	1.10 (0.97, 1.24)
Simple reaction time variability	1.20 (1.05, 1.38)	1.19 (1.03, 1.37)	1.22 (1.06, 1.41)	1.20 (1.04, 1.39)
Choice reaction time mean	0.95 (0.78, 1.16)	0.93 (0.76, 1.14)	0.98 (0.81, 1.20)	0.97 (0.80, 1.18)
Choice reaction time variability	0.98 (0.86, 1.12)	0.98 (0.86, 1.12)	1.00 (0.88, 1.13)	0.99 (0.87, 1.13)

* Pack years of smoking has been included as an additional confounder in model 3 for colorectal cancer.

**Table 4:** Hazard ratios and 95% confidence intervals for the relation of a one standard deviation increase (disadvantage) with cancer incidence (N = 6900).

	Model 1 (age and sex)	Model 2 (model 1 + social class)	Model 3 (model 1 + smoking, alcohol, physical activity)	Model 4 (all covariates)
**Prostate cancer (N = 3091, men only. cases = 99)**				
Simple reaction time mean	0.92 (0.74, 1.15)	0.92 (0.74, 1.15)	0.95 (0.77, 1.17)	0.95 (0.77, 1.17)
Simple reaction time variability	1.02 (0.83, 1.25)	1.02 (0.83, 1.26)	1.07 (0.86, 1.32)	1.07 (0.86, 1.33)
Choice reaction time mean	0.99 (0.77, 1.28)	1.00 (0.78, 1.29)	1.08 (0.85, 1.38)	1.10 (0.87, 1.40)
Choice reaction time variability	0.98 (0.85, 1.12)	0.98 (0.86, 1.13)	0.99 (0.87, 1.13)	1.00 (0.87, 1.13)
**Female breast cancer (N = 3809, women only. cases = 142)**				
Simple reaction time mean	1.02 (0.86, 1.22)	1.03 (0.87, 1.22)	1.02 (0.86, 1.21)	1.03 (0.87, 1.22)
Simple reaction time variability	0.99 (0.81, 1.20)	1.01 (0.83, 1.23)	1.00 (0.82, 1.21)	1.01 (0.83, 1.23)
Choice reaction time mean	1.08 (0.87, 1.34)	1.10 (0.88, 1.37)	1.12 (0.89, 1.39)	1.13 (0.90, 1.41)
Choice reaction time variability	1.03 (0.85, 1.25)	1.04 (0.86, 1.27)	1.04 (0.86, 1.27)	1.05 (0.86, 1.27)

## Discussion

In the present study, we found no clear pattern of association of simple and choice reaction time with cancer of all sites combined or specific cancer type. In the HALS sample, after controlling for age and sex, only lung cancer, colorectal cancer and skin cancer were significantly related to choice reaction time mean (longer choice reaction times were associated with elevated risk of lung cancer) and simple reaction time variability (greater variability in simple reaction times were associated with elevated risk of skin cancer) respectively after 25 years of follow-up. However, after the inclusion of additional covariates (social class initially), the results were reduced to non-significance for lung cancer. Simple reaction time variability was also significantly associated with colorectal cancer after controlling for age, sex and occupational social class to the same degree as lung cancer (HR = 1.20). For the association between simple reaction time variability and skin cancer, the effect remained after controlling for occupational social class, smoking, alcohol and physical activity. However, this result was counter to previous literature as more variable reaction time scores was associated with an increased risk of skin cancer. There was no association between any of the reaction time measures and all cancer, all smoking-related cancers, all non smoking-related cancers, prostate cancer and female breast cancer.

No study to date has examined the relation between reaction time and cancer incidence; rather, they have focused on more standard tests of cognition and reported either cancer mortality [23], or grouped mortality and morbidity together [20], [21]. Results of these studies have been mixed. Hart [20] reported a higher cause-specific mortality or cancer incidence risk with lower IQ for all-cancers, lung cancer and stomach cancer, and a lower incidence of colorectal and female breast cancer. However, the relationships were only statistically significant for lung cancer and, after full adjustment for confounding variables, none of the cancers was significantly related to IQ. This mirrors the results also found here for lung cancer. Furthermore, Shipley [3], also using this same HALS sample, reported no association between lung and all non-lung cancer mortality and cognition. Adding to these null results is Batty [21] who studied a cohort of one million Swedish men. They found modest but non-significant associations for cognition and lung, stomach, oesophageal and liver cancer. However, skin cancer showed a significant positive association. For each standard deviation increase in IQ advantage, the risk of skin cancer increased (HR = 1.18). The result remained after controlling for markers of socioeconomic position. In the current study, a significant association for skin cancer with reaction time variability was also noted (HR = 1.20 after controlling for all covariates). However, it ran contrary to this previous finding by Batty et al [21]. Here, more variable reaction times were associated with an elevated risk of skin cancer. Deary [14] then examined the association between IQ at age 11 and survival to age 76 in a follow up of the Scottish Mental Survey 1932 and reported that the risk of dying from lung and stomach cancers was significantly associated with lower childhood IQ scores. In support of these findings, Batty [23] utilized the Vietnam Experience Study and reported an inverse significant relation between age 20 IQ and death due to all cancers. Many of these findings mirror the findings in the current study, although we used reaction time as a measure of brain processing speed instead of the more standard cognitive performance measures. Therefore, taken together, the general trend of null results seen in Deary [21], Shipley [3] and Hart [20] suggest that in the United Kingdom-based studies, lower cognitive ability may be a risk factor for lung and stomach cancer mortality, but not cancer incidence. Further investigation is warranted.

One strength of the present study is that it is based on a population sample with an unrestricted age range of adults. Furthermore, cancer was grouped into site-specific malignancies as well as examining all cancers. As cancer has differing aetiologies at different anatomical locations, distinct site-specific malignancies may be associated with cognition in different ways. Also, reaction time was the measure of cognition used, which has been shown to be a valid, culturally-reduced measure of the underlying processes of cognition; i.e., speed of information processing [24], [25].

One weakness of the present study is that other potential risk factors for cancer, such as exposure to biological carcinogens, physical carcinogens and genetic predispositions, were not measured and consequently not controlled for in the analysis. Also, the long follow-up period of 25 years for cancer registrations from the baseline assessment of reaction time and the covariates could have led to fluctuations in these predictor and exposure variables. These unaccounted-for changes in social class, smoking, alcohol and physical activity and possibly reaction time (had the tests been completed several years later) affect the analyses results and their interpretations. Unfortunately, this problem was unavoidable for HALS as no further data collection had been completed.

As mentioned above we have reported this data in previous papers [2], [3]. However this is the first paper where we have used reaction time as the exposure. Using data from HALS, the current study suggests that slower simple or choice reaction time is not significantly associated with later all cancer incidence or site-specific cancer incidence. Exceptions to this may be lung cancer, colorectal cancer and skin cancer. However, the skin cancer result runs contrary to previous literature and so requires further investigation to determine whether it is a true result or a type 1 error.
